# Respiratory Syncytial Virus and Other Respiratory Viruses in Hospitalized Infants During the 2023–2024 Winter Season in Mexico

**DOI:** 10.3390/v16121917

**Published:** 2024-12-14

**Authors:** José J. Leija-Martínez, Sandra Cadena-Mota, Ana María González-Ortiz, Juan Carlos Muñoz-Escalante, Gabriel Mata-Moreno, Pedro Gerardo Hernández-Sánchez, María Vega-Morúa, Daniel E. Noyola

**Affiliations:** 1 Infectious Diseases Laboratory, Centro de Investigación en Ciencias de la Salud y Biomedicina, Universidad Autónoma de San Luis Potosí, San Luis Potosi 78210, Mexico; jesus.leija@uaslp.mx (J.J.L.-M.); carlos.escalante@uaslp.mx (J.C.M.-E.);; 2 Microbiology Department, Facultad de Medicina, Universidad Autónoma de San Luis Potosí, San Luis Potosi 78210, Mexico; sandra.cadena@uaslp.mx; 3 Hospital del Niño y la Mujer “Dr. Alberto López Hermosa”, San Luis Potosi 78364, Mexico; anagon71@yahoo.com.mx (A.M.G.-O.);; 4 Histocompatibility Laboratory, Centro de Investigación en Ciencias de la Salud y Biomedicina, Universidad Autónoma de San Luis Potosí, San Luis Potosi 78210, Mexico; gabriel.mata@uaslp.mx

**Keywords:** respiratory syncytial virus, severe acute respiratory syndrome coronavirus type 2, human metapneumovirus, influenza, acute respiratory infections, hospitalization, mortality, Mexico

## Abstract

Respiratory syncytial virus (RSV) is the leading cause of lower respiratory tract infections in young children. During the COVID-19 pandemic, a significant change in the epidemiology of RSV and other viruses occurred worldwide, leading to a reduction in the circulation of these infectious agents. After the pandemic, the resurgence of seasonal respiratory viruses occurred, but some features of these infections contrast to those registered prior to the pandemic. In the present work, we studied 390 children <5 years old admitted to the hospital to determine the contribution of RSV, SARS-CoV-2, human metapneumovirus (hMPV), and influenza viruses to acute respiratory infections during the 2023–2024 winter season in Mexico. RSV was the most frequently detected virus (n = 160, 41%), followed by SARS-CoV-2 (n = 69, 17.7%), hMPV (n = 68, 17.4%), and influenza A or B (n = 40, 10.26%). Fourteen patients required admission to the intensive care unit, including six (42.8%) with RSV infection. Four children died (1%). At least one of the four viruses was detected in all deceased patients: SARS-CoV-2 in one; SARS-CoV-2 and hMPV in two; and RSV, influenza A, and SARS-CoV-2 in one. The high impact of RSV and other respiratory viruses indicates the need to implement specific preventive programs to reduce the morbidity and mortality associated with them.

## 1. Introduction

Acute respiratory infections are one of the leading causes of morbidity and mortality worldwide, particularly in children [1]. While the etiology of these infections is wide, respiratory viruses are the leading cause of lower respiratory tract infections (LRTIs) in infants. Respiratory syncytial virus (RSV) is the most commonly recognized cause of LRTIs worldwide [2]. In 2019, there were an estimated 33.0 million RSV-related LRTIs, 3.6 million RSV-related LRTI hospital admissions, and 26,300 RSV-related LRTI in-hospital deaths [2]. In addition, human metapneumovirus (hMPV), influenza, and other viruses also contribute significantly to LRTI-associated hospitalizations in children [3,4]. Moreover, the SARS-CoV-2 pandemic has underscored the contribution of emerging viruses as causes of hospitalizations and deaths.

The role of respiratory viruses as leading causes of hospital admissions in children has been established for a long time. For example, a systematic review reported that the prevalence of RSV in children younger than two years with bronchiolitis was 59.2% (95% CI [54.7; 63.6]), making it the most common respiratory virus in that study [5]. In addition, during the pre-COVID-19 period, Pratt et al. [6] documented an RSV prevalence of 22.7% (95% CI [20.9; 24.5]) in community-acquired pneumonia in children younger than 18 years. Similarly, an RSV prevalence of 23% (95% CI [20.0; 25.0]) in children younger than 18 years with acute respiratory illnesses in Africa has been reported [7]. Influenza and human metapneumovirus (hMPV) are also important pathogens associated with severe LRTI in children [4,8].

Of note, the SARS-CoV-2 pandemic was associated with major changes in the epidemiology of other viruses. During the 2020–2021 winter season, the circulation of RSV, influenza, and several other viruses, was almost absent worldwide [9]. This has been attributed largely to the institution of non-pharmacological interventions to reduce SARS-CoV-2 transmission [10]. Of note, once social-distancing and reduced mobility recommendations were eased, there was an increase in circulation of non-SARS-CoV-2 viruses. As such, the continued monitoring and analysis of the epidemiological behavior of respiratory viruses is crucial to understanding the effects of emerging viruses and of interventions established to control the epidemics associated with them.

Previous studies have shown that during the 2021–2022 and 2022–2023 winter seasons, several changes in the epidemiology of RSV and other respiratory viruses have occurred [9,10]. The two most notable characteristics noted are (1) changes in the age group distribution of infants presenting with severe infections (i.e., requiring hospitalization) [11] and (2) changes in the seasonality of outbreaks caused by RSV and other viruses [12]. In the present study, we analyzed the etiology, as well as clinical and epidemiological characteristics, of LRTI in children <5 years of age hospitalized during the 2023–2024 winter season in San Luis Potosí, Mexico. Our results show that, while respiratory infections had an overall pattern resembling the one occurring before the SARS-CoV-2 pandemic, some epidemiological changes observed in association with the pandemic are still evident. An analysis of the current prevalence, clinical features, and outcomes of respiratory viruses should be helpful to inform public health strategies and clinical management of patients affected by these pathogens.

## 2. Methods

### 2.1. Study Site and Subjects

A prospective cross-sectional study of hospitalized children under five years of age with LRTIs was conducted from 19 August 2023 to 16 August 2024 (from epidemiological week 33 of 2023 to the epidemiological week 32 of 2024) at Hospital del Niño y la Mujer “Dr. Alberto López Hermosa” in San Luis Potosí City, Mexico.

Children under five years old with LRTIs who were hospitalized were included. The enrollment was carried out in the pediatric wards, the emergency department, and intensive care unit. Symptoms of LRTIs in these participants included nasal congestion, sneezing, rhinorrhea, cough, shortness of breath, difficulty breathing, chest retractions, wheezing, crackles, cyanosis, and apnea. Additionally, gastrointestinal and systemic symptoms such as fever, poor feeding, lethargy, diarrhea, and vomiting were registered. The diagnosis of bronchiolitis was recorded in participants with wheezing and rhonchi on auscultation, increased aeration, or peribronchial infiltration on chest X-rays. Infants with pneumonia had crackles on auscultation, and chest radiographs showed lung infiltration, lobar lung infiltration, pleural effusion, pneumothorax, and atelectasis. Participants with a barking cough, stridor, hoarseness, respiratory distress, fever, runny nose, and nasal congestion were diagnosed with laryngotracheobronchitis. Prolonged, repeated, or violent coughing episodes (paroxysms) and a whooping sound when inhaling after coughing stops were considered indicative of suspected pertussis. Pediatric patients admitted because of non-respiratory disorders who developed respiratory tract infections while in the hospital were considered to have nosocomial respiratory infections and were excluded from this study.

This study was approved by the Research and Ethics Committee of the Ministry of Health of the Government of the State of San Luis Potosí, Mexico, on 3 August 2023, with approval number SLP/04-2023. Study procedures adhered to the Declaration of Helsinki (2013) and were conducted following the Mexican Regulation for Health Research [13]. Before any procedures, each participant’s parents signed an informed consent form.

### 2.2. Viral Detection Assays

RNA was extracted from 140 µL of sample using the QIAamp Viral RNA Mini Kit (QIAGEN, Hilden, Germany), according to the manufacturer’s protocol. Purified RNA was stored at −70 °C until use. cDNA was synthetized using the RevertAid H Minus First Strand cDNA Synthesis Kit (Thermo Scientific, Waltham, MA, USA).

The detection of RSV, influenza A, influenza B, hMPV and SARS-CoV-2 was performed by qPCR from cDNA using the Maxima SYBR Green/Rox qPCR Master Mix (Thermo Scientific, Waltham, MA, USA) in a CFX Opus 96 Real-Time PCR System (Hercules, CA, USA). Samples were considered positive for each of the evaluated viruses if qPCR Cqs were below 38. The primers and thermocycling programs used for viral detection are described in Table 1 and Table 2.

**Table 1:** Primers used for detection of respiratory viruses by qPCR.

Primer ID	Target	Sequence	Reference
MCVF	RSV “P-M” region	5′-GGC AAA TAT GGA AAC ATA CGT GAA-3′	Lara-Hernandez et al. [14]
MCVR	RSV “P-M” region	5′-TCT TTT TCT AGG ACA TTG TAY TGA ACA-3′	
FLUAM-7-F	Influenza A “M2” gene	5′-CTT CTA ACC GAG GTC GAA ACG TA-3′	Terrier et al. [15]
FLUAM-161-R	Influenza A “M2” gene	5′-GGT GAC AGG ATT GGT CTT GTC TTT A-3′	
FLUBHA-940-F	Influenza B “HA” gene	5′-AAA TAC GGT GGA TTA AAC AAA AGC AA-3′	Van Elden et al. [16]
FLUBHA-1109-R	Influenza B “HA” gene	5′-CCA GCA ATA GCT CCG AAG AAA-3′	
NIID-N1-F	HMPV “N” gene	5′-TGA TGC RCT CAA AAG ATA CCC-3′	Sugimoto et al. [17]
NIID-N1-R	HMPV “N” gene	5′-ACR AAY AAA CTT TCT GCT TTG C-3′	
2019-nCoV_N1-F	SARS-CoV-2 “N” gene	5′-GAC CCC AAA ATC AGC GAA AT-3′	Centers for Disease Control and Prevention [18]
2019-nCoV_N1-R	SARS-CoV-2 “N” gene	5′-TCT GCT TAC TGC CAG TTG AAT CTG-3′	

This cut-off point was established based on protocols reported for the influenza virus and SARS-CoV-2; the primers used in this study are as reported by the World Health Organization and Centers for Disease Control and Prevention [18,19]. Based on the previous statement, we applied the same criteria for both RSV and hMPV detection.

### 2.3. Statistical Analysis

Clinical and demographic characteristics of patients were obtained from interviews with parents and reviews of medical records by a pediatrician. We compared the clinical features of participants with and without the detection of viral pathogens. In order to assess the difference between the pre-pandemic (2012–2015) and the post-pandemic (2023–2024) periods, we compared our results with the previous work by González-Ortiz et al. [20] in the same hospital. Quantitative data are presented as the median and interquartile range (IQR). To calculate the prevalence of viral detection, we divided the number of virus-positive samples by the total number of samples tested. Furthermore, confidence intervals at 95% (95% CI) for each proportion were calculated using binomial exact calculation. We conducted the Mann–Whitney U test to analyze significant differences between the medians of two groups and used the Kruskal–Wallis test for comparisons involving more than two groups. Proportions were compared using Fisher’s exact and Pearson’s chi-squared test as appropriate. All data were assessed using IBM^®^ SPSS^®^ Statistics v. 23.0 (SPSS, Inc., IBM, Chicago, IL, USA). In all instances, a *p*-value of less than 0.05 was considered as statistically significant.

## 3. Results

A total of 390 infants were included in the study during the one-year period. Admission diagnoses were pneumonia in 284 (72.8%), bronchiolitis in 90 (23.1%), pertussis-like syndrome in 11 (2.8%), and laryngotracheobronchitis in 5 (1.3%). The majority (n = 209, 53.4%) were younger than 12 months of age, and 227 (58.2%) were male. Other demographic and clinical characteristics of the study patients are shown in Table 3. Infants with bronchiolitis tended to be younger than those with pneumonia; 76.6% of them were younger than 12 months of age, and 91.1% were younger than 24 months. In contrast, children with pneumonia tended to be older than those with bronchiolitis, and this diagnosis was frequent across all ages.

At least one virus was detected in 248 (63.6%) admissions. The most frequently detected virus was RSV (n = 160, 41% of patients), followed by SARS-CoV-2 (n = 69, 17.7%), hMPV (n = 68, 17.4%), and influenza A or B (n = 40, 10.26%). Viruses were detected as single pathogens in 173 (44.3%) patients, and as coinfections (either two or three pathogens) in 75 (19.2%). The frequency of each combination of codetection is presented in Table 4. RSV was more commonly detected as a single infection, in contrast to SARS-CoV-2, hMPV, and influenza viruses, which were more commonly detected in conjunction with another virus. To better understand the results of the simultaneous detection of two or more viruses, we compared the Cq values for each virus between samples in which that virus was detected as a single pathogen, with those in which another virus was also detected (Supplementary Materials Table S1). Of interest, no differences in mean Cq values were observed between samples with single detection and dual/triple viral detection for RSV, hMPV, and influenza; in contrast, SARS-CoV-2 single positive samples had lower mean Cq values compared with those in which other viruses were also detected (mean Cq 31.1 vs. 33.9, *p* = 0.006).

Among patients with influenza infections, both influenza A (n = 20) and influenza B (n = 21) were detected (these include one patient with the detection of both influenza A and B).

The most common symptoms among the children admitted to the hospital were cough (98.2%) and rhinorrhea (84.4%), and 97.4% presented signs of respiratory distress. Other signs and symptoms are described in Table 5. Overall, demographic, epidemiological, and clinical characteristics of the patients in which specific viruses were detected were similar to those with a negative result for that virus (Supplementary Materials Tables S2–S9). A few exemptions were noted in patients with RSV infection: children in whom RSV was detected were younger, were diagnosed with pneumonia less frequently, and had congenital heart disease (CHD) or Down syndrome diagnosis less frequently than RSV negative patients (Supplementary Materials Table S2); in contrast, the prevalence of CHD in children in whom influenza was detected was higher than in influenza negative patients (Supplementary Materials Table S8). Moreover, participants who tested positive for RSV had a significantly longer median length of hospital stay than RSV-negative patients (5.5 days vs. 4.0 days, *p* = 0.008). These findings suggest that RSV infection is associated with prolonged hospitalization (Supplementary Materials Table S2).

Fourteen patients (3.6%) were admitted to the intensive care unit (ICU). No major differences were observed in the clinical features of children admitted to the ICU compared with those who were admitted to hospital wards only (Table 6). A virus was detected in nine (64.3%) of them: RSV was detected in six (42.8%), hMPV in four (28.6%), influenza type A in three (21.4%), and SARS-CoV-2 in two (14.3%) (including two patients with the simultaneous detection of two viruses and two patients with the simultaneous detection of three viruses), (Table 6). Four infants (1%) died. All of them had at least one virus detected: SARS-CoV-2 in one; SARS-CoV-2 and hMPV in two; and RSV, influenza A, and SARS-CoV-2 in one.

Comparisons between the children hospitalized with each virus showed several significant differences (Supplementary Materials Table S10); for these analyses, only patients in which a single virus was detected were included, in order to avoid duplicating data in cases with coinfection. Patients with SARS-CoV-2 were younger (median age 6.5 months) compared with those with hMPV, influenza, and RSV (*p* = 0.048). Additionally, CHD was more prevalent in influenza and SARS-CoV-2 patients (*p* = 0.001). Asthma was significantly associated with influenza infections (*p* = 0.022), and prematurity was higher among influenza and SARS-CoV-2 groups (*p* = 0.028). Down syndrome was more frequent in patients with hMPV and influenza infections, although no statistically significant differences were registered, compared with RSV and SARS-CoV-2. In contrast, the prevalence of symptoms among all participants with positive PCR results by virus group was practically the same, with no significant differences observed (Supplementary Materials Table S11).

Influenza vaccination was recorded in 96 (36.9%) of the 260 children older than 6 months of age (in whom this vaccine is indicated in Mexico). In contrast, SARS-CoV-2 vaccination is not recommended for children <5 years in Mexico and, therefore, none of the children were immunized against this virus. Palivizumab was not available at Hospital del Niño y la Mujer “Dr. Alberto López Hermosa” during the study period.

Our analysis indicates that the proportion of RSV-positive cases was 41.0% in the post-pandemic period (2023–2024) compared with 43.7% in the pre-pandemic period (2012–2015) (*p* = 0.35) (Supplementary Materials Table S12). Nevertheless, post-pandemic data (2023–2024) revealed a substantial shift in the age distribution of LRTIs (Supplementary Materials Table S13), with fewer infants under 12 months and more children aged 36 to 60 months affected, compared to the pre-pandemic period (*p* < 0.001). Specifically, RSV infections during the post-pandemic period showed a small, albeit significant, reduction in the proportion of children aged 0 to 6 months (*p* < 0.049), compared with findings in 2012–2015, as shown in Supplementary Materials Table S14.

Bacterial cultures (blood cultures or bronchoalveolar lavage) were rarely performed on participants in the study. Therefore, we could not determine the proportion of patients who had bacterial pneumonia. *Bordetella pertussis* PCR was carried out in six children with suspected pertussis, and it was positive in one.

## 4. Discussion

Once the dramatic impact of the SARS-CoV-2 pandemic has subsided, it is of great importance to establish the role that this virus will have, in conjunction with seasonal respiratory viruses. In the present study, we provide systematically obtained information regarding the contribution of major viruses to hospitalization in Mexican children during the 2023–2024 winter season.

RSV was the most frequently detected virus in this study. This virus has been consistently reported as the leading cause of LRTI in young children [2,3]. However, during the COVID-19 pandemic, major changes in the epidemiology of respiratory viruses occurred, with a notable reduction in RSV-associated hospitalizations during the 2020–2021 winter season, followed by an increase in severe cases during the following years [10]. In addition, changes in the epidemiological features of other respiratory viruses also occurred [9]. The present study showed that the contribution of RSV to hospitalizations in a pediatric population in Mexico is similar to that observed prior to the COVID-19 pandemic, with 41% of LRTI hospitalizations caused by this virus, a proportion similar to that registered at the same study hospital (43.7%) between 2012 and 2015 [19]. In addition, the seasonal distribution of RSV during the 2023–2024 season was similar to that observed before the COVID-19 pandemic in our city [20,21]. In contrast, although most infants hospitalized with RSV infection were under one year of age, the proportion of children older than 12 months (37.5%) was greater than that observed prior to the pandemic (26.2%) [21]. As such, while the overall impact of RSV on LRTI in young children is currently similar to that observed before the surge of SARS-CoV-2, some of the clinical features, such as changes in the age distribution of children with severe infection, continue to be affected by the pandemic. In addition, RSV contributed to severe infections, with the detection of this virus in 42.8% of the children admitted to the ICU, and in one (25%) of the infants who died.

SARS-CoV-2 and hMPV were the second and third most commonly detected viruses, with an almost equal number of positive cases. Of note, one or two additional viruses were detected in most infections in which either of these viruses were present. Previous studies in which the role of coinfections by respiratory viruses has been assessed have provided contrasting results, with most studies indicating that infections in which two viruses are detected are not associated with a greater severity of disease [22,23]. As such, when two or more viruses are detected, it is possible that, in some instances, this finding represents sequential viral infections in which one of the viruses is responsible for the acute disease leading to medical consultation [22]. Our results indirectly support this possibility for SARS-CoV-2 detections, as average cycle thresholds for SARS-CoV-2 positive samples with no detection of another virus were significantly lower than those of samples in which another virus was detected.

Similar to RSV, hMPV infections were significantly reduced during the SARS-CoV-2 pandemic [12]. However, fewer studies have analyzed the contribution of this virus to LRTI after the pandemic, compared with those addressing RSV infections. Interestingly, once social distancing measures were reduced, the circulation pattern of hMPV infections resembled that of RSV, with an increase in the mean age of children requiring hospitalization with this virus [11,24].

Global analyses carried out before the SARS-CoV-2 pandemic reported that, with the use of molecular methods, influenza was identified in 7.4% of LRTI hospitalizations in children <5 years [4]. Similarly, a study including patients from the present study site between 2014 and 2015 registered a 5.9% prevalence of influenza using molecular detection methods [25]. Globally, the COVID-19 pandemic generated unprecedented disruptions in influenza circulation, being particularly low between 2020 and 2021, showing an out-of-season resurgence in early 2022 [26]. Through 2023, these atypical patterns persisted, evidenced by late seasons in the Northern Hemisphere, early seasons in the Southern Hemisphere, and an increase in the inter-seasonal activity of influenza cases [27]. Our results indicate a slight increase in influenza prevalence compared to pre-pandemic data (10.26% vs. 5.9%); of note, fewer studies have compared the information on epidemiological influenza shifts in LRTI patterns before and after the COVID-19 pandemic than those comparing the post-COVID-19 era with the first two years of SARS-CoV-2 circulation [28,29].

Overall, our results indicate that RSV and other respiratory viruses remain as the leading causes of severe LRTI in Mexico. Influenza immunization has been included in the childhood immunization program in this country for over 20 years, and epidemiological evidence suggests that this has led to a reduction in severe infections caused by this virus [30,31]. However, the frequency of influenza vaccination in our study population was low. In addition, SARS-CoV-2 vaccination is not included in the immunization schedule for children in Mexico at present. Therefore, the strengthening of current immunization programs, and the development of additional programs targeting other viruses, is required in order to reduce the impact of these viruses in the infant population.

Overall, the most frequent underlying conditions in children hospitalized with LRTIs were prematurity (21%), asthma (7.4%), bronchopulmonary dysplasia (6.4%), and CHD (5.4%). Of note, the frequency of some of these conditions varied according to the virus associated with the infection. CHD and Down syndrome were less frequently found in patients with an RSV infection compared with those who tested negative for this virus. This contrasts with the established evidence indicating that children with Down syndrome and CHD have a significantly higher risk of severe RSV infection and hospitalization [32]. In contrast, CHD and Down syndrome were more frequent in children with influenza and SARS-CoV-2 infections. Of note, while both CHD and Down syndrome are well-established risk factors for severe RSV infections, they have also been associated with severe influenza and SARS-CoV-2 infections [33,34,35]. This may explain, in part, the aforementioned findings. In addition, the relatively small number of patients with Down syndrome and CHD may also limit the generalizability of our findings.

A limitation in our study is that diagnoses were based on admission diagnoses recorded by the treating physician for each patient. As a result, diagnoses were not established by a single person, but this depended on the hospital’s personnel, and differences in diagnostic criteria may have occurred as a result of the assessment of each physician at the time of admission. This had a particular impact on the diagnosis of bronchiolitis, since eight (8.9%) of the infants with this diagnosis were older than 24 months of age. Current guidelines limit the diagnosis of bronchiolitis to infants younger than 24 months, particularly those younger than 12 months [36]. However, bronchiolitis is still diagnosed by many physicians in older infants [37,38]. For instance, 13.8% of infants diagnosed with bronchiolitis at children’s hospitals in the United States between 2015 and 2022 were 24 to 59 months old [37]. As such, the application of standardized definitions should help to better understand the epidemiology of respiratory infections in the future. Also, we could not establish the contribution of bacterial pathogens to LRTIs, since bacterial cultures are not routinely carried out at the study site. *Bordetella pertussis* was detected by PCR in one child. However, the testing for this pathogen was carried out in only six children and, therefore, the contribution of this pathogen to LRTI could not be ascertained.

In conclusion, once the SARS-CoV-2 pandemic has subsided, this virus has started to present with a seasonal pattern, causing hospitalizations in children, together with other viruses. RSV persists as the leading cause of severe LRTI in Mexican children, accounting for 41% of admissions and contributing to 42.8% of ICU admissions. Infections with SARS-CoV-2, hMPV and influenza also accounted for a significant number of cases. Therefore, the strengthening of current immunization programs, as well as the development of new preventive strategies, are required to reduce the impact of these viruses in child morbidity and mortality.

## Figures and Tables

**Table 2:** Thermal-cycling conditions for detection of respiratory viruses by qPCR.

Virus	Denaturation Time	Annealing Temperature	Annealing Time	Number of Cycles
RSV	15 s	54.5 °C	30 s	40
Influenza A	15 s	60 °C	30 s	40
Influenza B	15 s	60 °C	30 s	40
hMPV	15 s	54.5 °C	30 s	40
SARS-CoV-2	30 s	55 °C	30 s	45

**Table 3:** Characteristics of infants <5 years admitted with LRTI during the 2023–2024 season.

Characteristic	Total (n = 390)
**Sex**	
Female, n (%)	163 (41.8%)
Male, n (%)	227 (58.2%)
**Age, months (median, IQR)**	11.0 (3.0–19.0)
**Age group (months)**	
Less than 6, n (%)	130 (33.3%)
6 to less than 12, n (%)	79 (20.3%)
12 to less than 24, n (%)	98 (25.1%)
24 to less than 36, n (%)	30 (7.7%)
36 to 60, n (%)	53 (13.6%)
**Diagnosis at Admission**	
Community-acquired pneumonia, n (%)	284 (72.8%)
Bronchiolitis, n (%)	90 (23.1%)
Pertussis-like syndrome, n (%)	11 (2.8%)
Laryngotracheobronchitis, n (%)	5 (1.3%)
**Underlying Conditions**	
Prematurity (<37 weeks), n (%)	82 (21.0%)
Asthma, n (%)	29 (7.4%)
Bronchopulmonary dysplasia, n (%)	25 (6.4%)
Congenital heart disease, n (%)	21 (5.4%)
Down syndrome, n (%)	10 (2.6%)
Immunodeficiency, n (%)	1 (0.3%)
**Environmental Conditions**	
Breastfeeding, n (%)	311 (79.7%)
Parental smoking, n (%)	109 (27.9%)
Wood usage for cooking, n (%)	36 (9.2%)
Siblings <5 years old, n (%)	149 (38.2%)
Number of siblings <5 years old (median, IQR)	1.0 (1.0–1.0)
Number of household members (median, IQR)	5.0 (4.0–6.0)
Daycare attendance, n (%)	30 (7.7%)
Exposure to persons with similar symptoms, n (%)	225 (57.7%)
**Severity Indicators**	
Length of hospital stay, days (median, IQR)	5.0 (2.3–7.0)
ICU admission, n (%)	14 (3.6%)
Mechanical ventilation, n (%)	16 (4.1%)
Death, n (%)	4 (1.0%)

**Table 4:** Viruses detected in children <5 years old admitted with lower respiratory tract infections.

Viral Identification	Total (n = 390)	RSV (Single or Codetection) (n = 160)	hMPV (Single or Codetection) (n = 68)	SARS-CoV-2 (Single or Codetection) (n = 69)	Influenza (Single or Codetection (n = 40)
RSV	98 (25.1%)	98 (61.3%)			
hMPV	31 (7.9%)		31 (45.6%)		
SARS-CoV-2	26 (6.7%)			26 (37.7%)	
Influenza	18 (4.6%)				18 (45%)
RSV + SARS-CoV-2	23 (5.9%)	23 (14.4%)		23 (33.3%)	
RSV + hMPV	18 (4.6%)	18 (11.3%)	18 (26.5%)		
RSV + Influenza	8 (2.1%)	8 (5%)			8 (20%)
hMPV + SARS-CoV-2	5 (1.3%)		5 (7.4%)	5 (7.2%)	
hMPV + Influenza	5 (1.3%)		5 (7.4%)		5 (13%)
SARS-CoV-2 + Influenza	2 (0.5%)			2 (2.9%)	2 (5%)
RSV + hMPV + SARS-CoV-2	7 (1.8%)	7 (4.4%)	7 (10.3%)	7 (10.1%)	
RSV + hMPV + Influenza	1 (0.3%)	1 (0.6%)	1 (1.5%)		1 (3%)
RSV + SARS-CoV-2 + Influenza	5 (1.3%)	5 (3.1%)		5 (7.2%)	5 (13%)
hMPV + SARS-CoV-2 + Influenza	1 (0.3%)		1 (1.5%)	1 (1.4%)	1 (3%)
Negative	142 (36.4%)				
**Total**	390 (100%)	160 (100%)	68 (100%)	69 (100%)	40 (100%)

Abbreviations: RSV—respiratory syncytial virus; hMPV—human metapneumovirus; SARS-CoV-2—severe acute respiratory syndrome coronavirus 2.

**Table 5:** Signs and symptoms of children admitted with LRTI.

Signs and Symptoms	Total (n = 390)
Cough, n (%)	383 (98.2%)
Rhinorrhea, n (%)	329 (84.4%)
Nasal Congestion, n (%)	322 (82.6%)
Fever, n (%)	273 (70.0%)
Sneezing, n (%)	231 (59.2%)
Vomiting, n (%)	133 (34.1%)
Diarrhea, n (%)	73 (18.7%)
Cyanosis, n (%)	69 (17.7%)
Apnea, n (%)	21 (5.4%)
Respiratory distress, n (%)	380 (97.4%)
Crackles, n (%)	310 (79.5%)
Wheezing, n (%)	188 (48.2%)

**Table 6:** Demographic and clinical characteristics by intensive care unit (ICU).

Characteristics	ICU Admission (n = 14)	No-ICU Admission (n = 376)	Total (n = 390)	*p*-Value
**Sex**				0.526
Female, n (%)	7 (50.0%)	156 (41.5%)	163 (41.8%)	
Male, n (%)	7 (50.0%)	220 (58.5%)	227 (58.2%)	
**Age, months, median (IQR)**	5.5 (2.0–12.0)	11.0 (4.0–19.0)	11.0 (3.0–19.0)	0.275 *
**Age Category (months)**				0.707
Less than 6, n (%)	7 (50.0%)	123 (32.7%)	130 (33.3%)	
6 to less than 12, n (%)	2 (14.3%)	77 (20.5%)	79 (20.3%)	
12 to less than 24, n (%)	2 (14.3%)	96 (25.5%)	98 (25.1%)	
24 to less than 36, n (%)	1 (7.1%)	29 (7.7%)	30 (7.7%)	
36 to 60, n (%)	2 (14.3%)	51 (13.6%)	53 (13.6%)	
**Diagnosis at Admission**				0.144
Community-acquired pneumonia, n (%)	14 (100.0%)	270 (71.8%)	284 (72.8%)	
Bronchiolitis, n (%)	0 (0.0%)	90 (23.9%)	90 (23.1%)	
Pertussis-like syndrome, n (%)	0 (0.0%)	11 (2.9%)	11 (2.8%)	
Laryngotracheobronchitis, n (%)	0 (0.0%)	5 (1.3%)	5 (1.3%)	
**Underlying Conditions**				
Congenital heart disease, n (%)	2 (14.3%)	19 (5.1%)	21 (5.4%)	0.133
Down syndrome, n (%)	0 (0.0%)	10 (2.7%)	10 (2.6%)	0.536
Bronchopulmonary dysplasia, n (%)	2 (14.3%)	23 (6.1%)	25 (6.4%)	0.22
Immunodeficiency, n (%)	0 (0.0%)	1 (0.3%)	1 (0.3%)	0.847
Asthma, n (%)	1 (7.1%)	28 (7.4%)	29 (7.4%)	0.966
Prematurity (<37 weeks), n (%)	3 (21.4%)	79 (21.0%)	82 (21.0%)	0.97
**Environmental Conditions**				
Breastfeeding, n (%)	9 (64.3%)	302 (80.3%)	311 (79.7%)	0.143
Parental smoking, n (%)	3 (21.4%)	106 (28.2%)	109 (27.9%)	0.58
Wood usage for cooking, n (%)	3 (21.4%)	33 (8.8%)	36 (9.2%)	0.108
Siblings <5 years old, n (%)	2 (14.3%)	147 (39.1%)	149 (38.2%)	0.061
Number of siblings <5 years, median (IQR)	1.0 (0.5–1.0)	1.0 (1.0–1.0)	1.0 (1.0–1.0)	0.027 *
Household size, median (IQR)	5.0 (4.0–5.5)	5.0 (4.0–6.0)	5.0 (4.0–6.0)	0.342 *
Daycare attendance, n (%)	0 (0.0%)	30 (8.0%)	30 (7.7%)	0.271
**Previous Hospitalizations**				
Previous hospitalizations, n (%)	5 (35.7%)	98 (26.1%)	103 (26.4%)	0.421
Number of previous hospitalizations, median (IQR)	1.0 (1.0–1.0)	1.0 (1.0–2.0)	1.0 (1.0–2.0)	0.811 *
**Virus status**				
RSV positive, n (%)	6 (42.9%)	154 (41.0%)	160 (41.0%)	0.887
hMPV positive, n (%)	4 (28.6%)	64 (17.0%)	68 (17.4%)	0.263
Influenza positive, n (%)	3 (21.4%)	37 (9.8%)	40 (10.3%)	0.161
SARS-CoV-2 positive, n (%)	2 (14.3%)	67 (17.8%)	69 (17.7%)	0.734

* Mann–Whitney U tests. Values are expressed as the medians and interquartile ranges. Abbreviations: IQR—Interquartile Range; RSV—respiratory syncytial virus; hMPV—human metapneumovirus; SARS-CoV-2—severe acute respiratory syndrome coronavirus 2.

## Data Availability

The study data of this study are available from the corresponding author, upon reasonable request.

## Supplementary Materials

The following supporting information can be downloaded at: https://www.mdpi.com/article/10.3390/v16121917/s1, Table S1. Comparison of Cq values for viral detection between samples, in which each virus was detected as a single pathogen or together with other viruses; Table S2. Demographic and clinical characteristics by RSV status; Table S3. Prevalence of symptoms by RSV status; Table S4. Demographic and clinical characteristics by human metapneumovirus status (hMPV); Table S5. Prevalence of symptoms by human metapneumovirus status (hMPV); Table S6. Demographic and clinical characteristics by SARS-CoV-2 status; Table S7. Prevalence of symptoms by severe acute respiratory syndrome coronavirus 2 (SARS-CoV-2) status; Table S8. Demographic and clinical characteristics by influenza status; Table S9. Prevalence of symptoms by influenza status; Table S10. Demographic and clinical characteristics among pediatric patients with positive PCR results by virus group; Table S11. Prevalence of symptoms among pediatric patients with positive PCR results by virus group; Table S12. Comparison of RSV positivity and negativity proportions between post-pandemic (2023–2024) and pre-pandemic (2012–2015) seasons; Table S13. Age category comparison between post-pandemic (2023–2024) and pre-pandemic (2012–2015) cohorts of lower respiratory tract infections; Table S14. Age category comparison between post-pandemic (2023–2024) and pre-pandemic (2012–2015) cohorts of lower respiratory tract infections related to respiratory syncytial virus.
