# Supplementary material for: Respiratory Syncytial Virus and Other Respiratory Viruses in Hospitalized Infants During the 2023–2024 Winter Season in Mexico
Source: Viruses. 2024 Dec 14;16(12):1917. doi: 10.3390/v16121917 (PMC11680223; doi:10.3390/v16121917)
Supplement: Supplementary file 1 [file viruses-16-01917-s001.zip › viruses-3338033-supplementary.pdf]

viruses

Respiratory Syncytial Virus and Other Respiratory Viruses in Hospitalized Infants During the 2023–2024 Winter Season in Mexico

SUPPLEMENTARY TABLES

Supplementary Table S1. Comparison of Cq values for viral detection between samples in which each virus was detected as single pathogen or together with other viruses.

Virus	Single virus (n)	Single virus (mean Cq)	Single virus (std dev Cq)	Coinfection (n)	Coinfection (mean Cq)	Coinfection (std dev Cq)	P value
RSV	98	23.9	6.3	62	24.7	6.4	0.49
hMPV	31	26.2	5.8	37	27.9	6.1	0.26
Influenza	18	33.0	4.2	22	32.4	4.1	0.63
SARS-CoV-2	26	31.1	5.8	43	33.9	2.1	0.006

Supplementary Table S2. Demographic and clinical characteristics by RSV status.

Characteristics	RSV positive (n=160)	RSV negative (n=230)	Total (n=390)	p-Value
**Sex**				0.057
Female, n (%)	76 (47.5%)	87 (37.8%)	163 (41.8%)	
Male, n (%)	84 (52.5%)	143 (62.2%)	227 (58.2%)	
**Age, months (median, IQR)**	8.0 (2.0 – 15.0)	12.0 (4.0 – 23.0)	11.0 (3.0 – 19.0)	0.003*
**Age Category (months)**				0.05
Less than 6, n (%)	64 (40.0%)	66 (28.7%)	130 (33.3%)	
6 to less than 12, n (%)	36 (22.5%)	43 (18.7%)	79 (20.3%)	
12 to less than 24, n (%)	30 (18.8%)	68 (29.6%)	98 (25.1%)	
24 to less than 36, n (%)	11 (6.9%)	19 (8.3%)	30 (7.7%)	
36 to 60, n (%)	19 (11.9%)	34 (14.8%)	53 (13.6%)	
**Diagnosis at Admission**				0.028
Bronchiolitis, n (%)	39 (24.4%)	51 (22.2%)	90 (23.1%)	
Laryngotracheobronchitis, n (%)	1 (0.6%)	4 (1.7%)	5 (1.3%)	
Community-acquired pneumonia, n (%)	111 (69.4%)	173 (75.2%)	284 (72.8%)	
Pertussis-like syndrome, n (%)	9 (5.6%)	2 (0.9%)	11 (2.8%)	
**Underlying Conditions**				
Congenital heart disease, n (%)	2 (1.2%)	19 (8.3%)	21 (5.4%)	0.003
Down syndrome, n (%)	1 (0.6%)	9 (3.9%)	10 (2.6%)	0.043
Bronchopulmonary dysplasia, n (%)	7 (4.4%)	18 (7.8%)	25 (6.4%)	0.171
Immunodeficiency, n (%)	0 (0.0%)	1 (0.4%)	1 (0.3%)	1.0
Asthma, n (%)	12 (7.5%)	17 (7.4%)	29 (7.4%)	0.968
Prematurity (<37 weeks), n (%)	33 (20.6%)	49 (21.3%)	82 (21.0%)	0.871
**Environmental Conditions**				
Breastfeeding, n (%)	133 (83.1%)	178 (77.4%)	311 (79.7%)	0.166
Parental smoking, n (%)	45 (28.1%)	64 (27.8%)	109 (27.9%)	0.948
Wood usage for cooking, n (%)	10 (6.2%)	26 (11.3%)	36 (9.2%)	0.09
Siblings <5 years old, n (%)	73 (45.6%)	76 (33.0%)	149 (38.2%)	0.012
Number of siblings <5 years old (median, IQR)	1.0 (1.0 – 1.0)	1.0 (1.0 – 1.0)	1.0 (1.0 – 1.0)	0.317*

Number of household members (median, IQR)	5.0 (4.0 – 6.0)	5.0 (4.0 – 6.0)	5.0 (4.0 – 6.0)	0.674*
Daycare attendance, n (%)	10 (6.2%)	20 (8.7%)	30 (7.7%)	0.373
Exposure to similar cases, n (%)	113 (70.6%)	112 (48.7%)	225 (57.7%)	<0.001
**Previous Hospitalizations**				
Previous hospitalizations, n (%)	28 (17.5%)	75 (32.6%)	103 (26.4%)	0.001
Number of previous hospitalizations (median, IQR)	1.0 (1.0 – 1.0)	1.0 (1.0 – 2.0)	1.0 (1.0 – 2.0)	0.913*
**Severity Indicators**				
Length of hospital stay, days (median, IQR)	5.5 (3.0 – 8.0)	4.0 (2.0 – 7.0)	5.0 (2.3 – 7.0)	0.008*
Intensive Care Unit admission, n (%)	6 (3.8%)	8 (3.5%)	14 (3.6%)	0.887
Mechanical ventilation, n (%)	5 (3.1%)	11 (4.8%)	16 (4.1%)	0.417
Death, n (%)	1 (0.6%)	3 (1.3%)	4 (1.0%)	0.914

*Mann-Whitney U tests. Values are expressed as the medians and interquartile ranges. Abbreviations: IQR, Interquartile Range.

Supplementary Table S3. Prevalence of symptoms by RSV status.

Signs and symptoms	RSV positive (n=160)	RSV negative (n=230)	Total (n=390)	p-Value
Fever, n (%)	111 (69.4%)	162 (70.4%)	273 (70.0%)	0.822
Wheezing, n (%)	71 (44.4%)	117 (50.9%)	188 (48.2%)	0.207
Crackles, n (%)	126 (78.8%)	184 (80.0%)	310 (79.5%)	0.764
Respiratory distress, n (%)	159 (99.4%)	221 (96.1%)	380 (97.4%)	0.043
Cyanosis, n (%)	31 (19.4%)	38 (16.5%)	69 (17.7%)	0.468
Cough, n (%)	158 (98.8%)	225 (97.8%)	383 (98.2%)	0.705
Apnea, n (%)	10 (6.2%)	11 (4.8%)	21 (5.4%)	0.528
Rhinorrhea, n (%)	144 (90.0%)	185 (80.4%)	329 (84.4%)	0.011
Nasal Congestion, n (%)	138 (86.2%)	184 (80.0%)	322 (82.6%)	0.11
Sneezing, n (%)	96 (60.0%)	135 (58.7%)	231 (59.2%)	0.797
Vomiting, n (%)	55 (34.4%)	78 (33.9%)	133 (34.1%)	0.925
Diarrhea, n (%)	24 (15.0%)	49 (21.3%)	73 (18.7%)	0.116

Supplementary Table S4. Demographic and clinical characteristics by human metapneumovirus status (hMPV).

Characteristics	hMPV positive (n=68)	hMPV negative (n=322)	Total (n=390)	p-Value
**Sex**				0.909
Female, n (%)	28 (41.2%)	135 (41.9%)	163 (41.8%)	
Male, n (%)	40 (58.8%)	187 (58.1%)	227 (58.2%)	
**Age, months (median, IQR)**	10.5 (4.25–24.0)	11.0 (3.0–18.25)	11.0 (3.0–19.0)	0.297*
**Age Category (months)**				0.505
Less than 6, n (%)	19 (27.9%)	111 (34.5%)	130 (33.3%)	
6 to less than 12, n (%)	16 (23.5%)	63 (19.6%)	79 (20.3%)	
12 to less than 24, n (%)	15 (22.1%)	83 (25.8%)	98 (25.1%)	
24 to less than 36, n (%)	5 (7.4%)	25 (7.8%)	30 (7.7%)	
36 to 60, n (%)	13 (19.1%)	40 (12.4%)	53 (13.6%)	
**Diagnosis at Admission**				0.211
Bronchiolitis, n (%)	9 (13.2%)	81 (25.2%)	90 (23.1%)	
Laryngotracheobronchitis, n (%)	1 (1.5%)	4 (1.2%)	5 (1.3%)	

Community-acquired pneumonia, n (%)	56 (82.4%)	228 (70.8%)	284 (72.8%)	
Pertussis-like syndrome, n (%)	2 (2.9%)	9 (2.8%)	11 (2.8%)	
**Underlying Conditions**				
Congenital heart disease, n (%)	2 (2.9%)	19 (5.9%)	21 (5.4%)	0.326
Down syndrome, n (%)	4 (5.9%)	6 (1.9%)	10 (2.6%)	0.057
Bronchopulmonary dysplasia, n (%)	2 (2.9%)	23 (7.1%)	25 (6.4%)	0.199
Immunodeficiency, n (%)	1 (1.5%)	0 (0.0%)	1 (0.3%)	0.174
Asthma, n (%)	7 (10.3%)	22 (6.8%)	29 (7.4%)	0.323
Prematurity (<37 weeks), n (%)	7 (10.3%)	75 (23.3%)	82 (21.0%)	0.017
**Environmental Conditions**				
Breastfeeding, n (%)	55 (80.9%)	256 (79.5%)	311 (79.7%)	0.797
Parental smoking, n (%)	21 (30.9%)	88 (27.3%)	109 (27.9%)	0.553
Wood usage for cooking, n (%)	5 (7.4%)	31 (9.6%)	36 (9.2%)	0.556
Siblings <5 years old, n (%)	26 (38.2%)	123 (38.2%)	149 (38.2%)	0.996
Number of siblings <5 years old (median, IQR)	1.0 (1.0–1.0)	1.0 (1.0–1.0)	1.0 (1.0–1.0)	0.707*
Number of household members (median, IQR)	5.0 (4.0–6.0)	5.0 (4.0–6.0)	5.0 (4.0–6.0)	0.756*
Daycare attendance, n (%)	4 (5.9%)	26 (8.1%)	30 (7.7%)	0.538
Exposure to similar cases, n (%)	44 (64.7%)	181 (56.2%)	225 (57.7%)	0.198
**Previous Hospitalizations**				
Previous hospitalizations, n (%)	22 (32.4%)	81 (25.2%)	103 (26.4%)	0.221
Number of previous hospitalizations (median, IQR)	1.0 (1.0–1.0)	1.0 (1.0–2.0)	1.0 (1.0–2.0)	0.205*
**Severity Indicators**				
Length of hospital stay, days (median, IQR)	5.5 (3.0–8.0)	4.0 (2.0–7.0)	5.0 (2.0–7.0)	0.072*
Intensive Care Unit admission, n (%)	4 (5.9%)	10 (3.1%)	14 (3.6%)	0.263
Mechanical ventilation, n (%)	3 (4.4%)	13 (4.0%)	16 (4.1%)	0.887
Death, n (%)	2 (2.9%)	2 (0.6%)	4 (1.0%)	0.234

*Mann-Whitney U tests. Values are expressed as the medians and interquartile ranges. Abbreviations: IQR, Interquartile Range.

Supplementary Table S5. Prevalence of symptoms by human metapneumovirus status (hMPV).

**Signs and Symptoms**	**hMPV positive (n=68)**	**hMPV negative (n=322)**	**Total (n=390)**	**p-Value**
Fever, n (%)	57 (83.8%)	216 (67.1%)	273 (70.0%)	0.006
Wheezing, n (%)	23 (33.8%)	165 (51.2%)	188 (48.2%)	0.009
Crackles, n (%)	57 (83.8%)	253 (78.6%)	310 (79.5%)	0.33
Respiratory distress, n (%)	68 (100.0%)	312 (96.9%)	380 (97.4%)	0.141
Cyanosis, n (%)	14 (20.6%)	55 (17.1%)	69 (17.7%)	0.491
Cough, n (%)	68 (100.0%)	315 (97.8%)	383 (98.2%)	0.22
Apnea, n (%)	2 (2.9%)	19 (5.9%)	21 (5.4%)	0.326
Rhinorrhea, n (%)	57 (83.8%)	272 (84.5%)	329 (84.4%)	0.894
Nasal Congestion, n (%)	58 (85.3%)	264 (82.0%)	322 (82.6%)	0.514
Sneezing, n (%)	33 (48.5%)	198 (61.5%)	231 (59.2%)	0.048
Vomiting, n (%)	27 (39.7%)	106 (32.9%)	133 (34.1%)	0.283
Diarrhea, n (%)	13 (19.1%)	60 (18.6%)	73 (18.7%)	0.926

Supplementary Table S6. Demographic and clinical characteristics by SARS-CoV-2 status.

Characteristics	SARS-CoV-2 Positive (n=69)	SARS-CoV-2 Negative (n=321)	Total (n=390)	p-Value
**Sex**				0.822
Female, n (%)	28 (40.6%)	135 (42.1%)	163 (41.8%)	
Male, n (%)	41 (59.4%)	186 (57.9%)	227 (58.2%)	
**Age, months (median, IQR)**	8.0 (2.5 – 16.0)	11.0 (4.0 – 20.0)	11.0 (3.0 – 19.0)	0.153*
**Age Category (months)**				0.193
Less than 6, n (%)	26 (37.7%)	104 (32.4%)	130 (33.3%)	
6 to less than 12, n (%)	18 (26.1%)	61 (19.0%)	79 (20.3%)	
12 to less than 24, n (%)	11 (15.9%)	87 (27.1%)	98 (25.1%)	
24 to less than 36, n (%)	7 (10.1%)	23 (7.2%)	30 (7.7%)	
36 to 60, n (%)	7 (10.1%)	46 (14.3%)	53 (13.6%)	
**Diagnosis at Admission**				0.457
Bronchiolitis, n (%)	13 (18.8%)	77 (24.0%)	90 (23.1%)	
Laryngotracheobronchitis, n (%)	0 (0.0%)	5 (1.6%)	5 (1.3%)	
Community-acquired pneumonia, n (%)	53 (76.8%)	231 (72.0%)	284 (72.8%)	
Pertussis-like syndrome, n (%)	3 (4.3%)	8 (2.5%)	11 (2.8%)	
**Underlying Conditions**				
Congenital heart disease, n (%)	5 (7.2%)	16 (5.0%)	21 (5.4%)	0.45
Down syndrome, n (%)	1 (1.4%)	9 (2.8%)	10 (2.6%)	0.518
Bronchopulmonary dysplasia, n (%)	5 (7.2%)	20 (6.2%)	25 (6.4%)	0.755
Immunodeficiency, n (%)	0 (0.0%)	1 (0.3%)	1 (0.3%)	0.642
Asthma, n (%)	6 (8.7%)	23 (7.2%)	29 (7.4%)	0.66
Prematurity (<37 weeks), n (%)	16 (23.2%)	66 (20.6%)	82 (21.0%)	0.627
**Environmental Conditions**				
Breastfeeding, n (%)	53 (76.8%)	258 (80.4%)	311 (79.7%)	0.504
Parental smoking, n (%)	17 (24.6%)	92 (28.7%)	109 (27.9%)	0.499
Wood usage for cooking, n (%)	2 (2.9%)	34 (10.6%)	36 (9.2%)	0.045
Siblings <5 years old, n (%)	26 (37.7%)	123 (38.3%)	149 (38.2%)	0.921
Number of siblings <5 years old (median, IQR)	1.0 (1.0 – 1.0)	1.0 (1.0 – 1.0)	1.0 (1.0 – 1.0)	0.760*
Number of household members (median, IQR)	5.0 (4.0 – 7.0)	5.0 (4.0 – 6.0)	5.0 (4.0 – 6.0)	0.340*
Daycare attendance, n (%)	9 (13.0%)	21 (6.5%)	30 (7.7%)	0.066
Exposure to similar cases, n (%)	41 (59.4%)	184 (57.3%)	225 (57.7%)	0.749
**Previous Hospitalizations**				
Previous hospitalizations, n (%)	17 (24.6%)	86 (26.8%)	103 (26.4%)	0.713
Number of previous hospitalizations (median, IQR)	1.0 (1.0 – 2.0)	1.0 (1.0 – 2.0)	1.0 (1.0 – 2.0)	0.458*
**Severity Indicators**				
Length of hospital stay, days (median, IQR)	4.0 (2.0 – 7.0)	5.0 (3.0 – 7.0)	5.0 (2.0 – 7.0)	0.433*
Intensive Care Unit admission, n (%)	2 (2.9%)	12 (3.7%)	14 (3.6%)	0.734
Mechanical ventilation, n (%)	2 (2.9%)	14 (4.4%)	16 (4.1%)	0.578
Death, n (%)	4 (5.8%)	0 (0.0%)	4 (1.0%)	0.002

*Mann-Whitney U tests. Values are expressed as the medians and interquartile ranges. Abbreviations: IQR, Interquartile Range.

Supplementary Table S7. Prevalence of symptoms by severe acute respiratory syndrome coronavirus 2 (SARS-CoV-2) status.

Signs and Symptoms	SARS-CoV-2 Negative (n=321)	SARS-CoV-2 Positive (n=69)	Total (n=390)	p-Value
Fever, n (%)	225 (70.1%)	48 (69.6%)	273 (70.0%)	0.931
Wheezing, n (%)	160 (49.8%)	28 (40.6%)	188 (48.2%)	0.162
Crackles, n (%)	253 (78.8%)	57 (82.6%)	310 (79.5%)	0.479
Respiratory distress, n (%)	311 (96.9%)	69 (100.0%)	380 (97.4%)	0.137
Cyanosis, n (%)	56 (17.4%)	13 (18.8%)	69 (17.7%)	0.783
Cough, n (%)	315 (98.1%)	68 (98.6%)	383 (98.2%)	0.812
Apnea, n (%)	16 (5.0%)	5 (7.2%)	21 (5.4%)	0.45
Rhinorrhea, n (%)	273 (85.0%)	56 (81.2%)	329 (84.4%)	0.42
Nasal Congestion, n (%)	263 (81.9%)	59 (85.5%)	322 (82.6%)	0.478
Sneezing, n (%)	194 (60.4%)	37 (53.6%)	231 (59.2%)	0.296
Vomiting, n (%)	108 (33.6%)	25 (36.2%)	133 (34.1%)	0.681
Diarrhea, n (%)	62 (19.3%)	11 (15.9%)	73 (18.7%)	0.515

Supplementary Table S8. Demographic and clinical characteristics by influenza status.

Characteristics	Influenza positive (n = 40)	Influenza negative (n = 350)	Total (n = 390)	p-Value
**Sex**				0.44
Female, n (%)	19 (47.5%)	144 (41.1%)	163 (41.8%)	
Male, n (%)	21 (52.5%)	206 (58.9%)	227 (58.2%)	
**Age, months (median, IQR)**	10.0 (3.0–17.3)	11.0 (3.8–20.0)	11.0 (3.0–19.0)	0.588*
**Age Category (months)**				0.336
Less than 6, n (%)	15 (37.5%)	115 (32.9%)	130 (33.3%)	
6 to less than 12, n (%)	6 (15.0%)	73 (20.9%)	79 (20.3%)	
12 to less than 24, n (%)	14 (35.0%)	84 (24.0%)	98 (25.1%)	
24 to less than 36, n (%)	1 (2.5%)	29 (8.3%)	30 (7.7%)	
36 to 60, n (%)	4 (10.0%)	49 (14.0%)	53 (13.6%)	
**Diagnosis at Admission**				0.561
Bronchiolitis, n (%)	7 (17.5%)	83 (23.7%)	90 (23.1%)	
Laryngotracheobronchitis, n (%)	0 (0.0%)	5 (1.4%)	5 (1.3%)	
Community-acquired pneumonia, n (%)	31 (77.5%)	253 (72.3%)	284 (72.8%)	
Pertussis-like syndrome, n (%)	2 (5.0%)	9 (2.6%)	11 (2.8%)	
**Underlying Conditions**				
Congenital heart disease, n (%)	5 (12.5%)	16 (4.6%)	21 (5.4%)	0.035
Down syndrome, n (%)	2 (5.0%)	8 (2.3%)	10 (2.6%)	0.304
Bronchopulmonary dysplasia, n (%)	4 (10.0%)	21 (6.0%)	25 (6.4%)	0.328
Immunodeficiency, n (%)	0 (0.0%)	1 (0.3%)	1 (0.3%)	1.0
Asthma, n (%)	5 (12.5%)	24 (6.9%)	29 (7.4%)	0.198
Prematurity (<37 weeks), n (%)	9 (22.5%)	73 (20.9%)	82 (21.0%)	0.809
**Environmental Conditions**				
Breastfeeding, n (%)	35 (87.5%)	276 (78.9%)	311 (79.7%)	0.198
Parental smoking, n (%)	15 (37.5%)	94 (26.9%)	109 (27.9%)	0.155
Gas stove usage, n (%)	38 (95.0%)	339 (96.9%)	377 (96.7%)	0.535
Wood stove usage, n (%)	4 (10.0%)	32 (9.1%)	36 (9.2%)	0.859
Siblings <5 years old, n (%)	16 (40.0%)	133 (38.0%)	149 (38.2%)	0.805
Number of siblings <5 years old (median, IQR)	1.0 (1.0–1.0)	1.0 (1.0–1.0)	1.0 (1.0–1.0)	0.641*

Number of household members (median, IQR)	5.0 (5.0–6.0)	5.0 (4.0–6.0)	5.0 (4.0–6.0)	0.832*
Daycare attendance, n (%)	1 (2.5%)	29 (8.3%)	30 (7.7%)	0.193
Exposure to similar cases, n (%)	22 (55.0%)	203 (58.0%)	225 (57.7%)	0.716
**Previous Hospitalizations**				
Previous hospitalizations, n (%)	11 (27.5%)	92 (26.3%)	103 (26.4%)	0.869
Number of previous hospitalizations (median, IQR)	1.0 (1.0–3.0)	1.0 (1.0–2.0)	1.0 (1.0–2.0)	0.778*
**Severity Indicators**				
Length of hospital stay, days (median, IQR)	5.0 (3.0–8.0)	4.5 (2.0–7.0)	5.0 (2.0–7.0)	0.494*
Intensive Care Unit admission, n (%)	3 (7.5%)	11 (3.1%)	14 (3.6%)	0.161
Mechanical ventilation, n (%)	2 (5.0%)	14 (4.0%)	16 (4.1%)	0.763
Death, n (%)	1 (2.5%)	3 (0.9%)	4 (1.0%)	0.705

*Mann-Whitney U tests. Values are expressed as the medians and interquartile ranges. Abbreviations: IQR, Interquartile Range.

Supplementary Table S9. Prevalence of symptoms by influenza status.

Signs and Symptoms	Influenza positive (n = 40)	Influenza negative (n = 350)	Total (n = 390)	p-Value
Fever, n (%)	30 (75.0%)	243 (69.4%)	273 (70.0%)	0.466
Wheezing, n (%)	15 (37.5%)	173 (49.4%)	188 (48.2%)	0.153
Crackles, n (%)	33 (82.5%)	277 (79.1%)	310 (79.5%)	0.618
Respiratory distress, n (%)	39 (97.5%)	341 (97.4%)	380 (97.4%)	0.978
Cyanosis, n (%)	8 (20.0%)	61 (17.4%)	69 (17.7%)	0.686
Cough, n (%)	40 (100.0%)	343 (98.0%)	383 (98.2%)	0.367
Apnea, n (%)	3 (7.5%)	18 (5.1%)	21 (5.4%)	0.532
Rhinorrhea, n (%)	29 (72.5%)	300 (85.7%)	329 (84.4%)	0.029
Nasal Congestion, n (%)	36 (90.0%)	286 (81.7%)	322 (82.6%)	0.191
Sneezing, n (%)	26 (65.0%)	205 (58.6%)	231 (59.2%)	0.433
Vomiting, n (%)	15 (37.5%)	118 (33.7%)	133 (34.1%)	0.632
Diarrhea, n (%)	11 (27.5%)	62 (17.7%)	73 (18.7%)	0.133

Supplementary Table S10. Demographic and clinical characteristics among pediatric patients with positive PCR results by virus group.

Variable	hMPV (n=31)	Influenza (n=18)	RSV (n=98)	SARS-CoV (n=26)	p-Value
**Sex**					0.495
Female, n (%)	11 (35.5%)	9 (50.0%)	48 (49.0%)	10 (38.5%)	
Male, n (%)	20 (64.5%)	9 (50.0%)	50 (51.0%)	16 (61.5%)	
**Age, months (median, IQR)**	12.0 (7.0–24.0)	12.5 (3.0–21.5)	10.0 (2.0–16.3)	6.5 (3.0–13.8)	0.048*
**Age Category (months)**					0.217
Less than 6, n (%)	6 (19.4%)	6 (33.3%)	40 (40.8%)	11 (42.3%)	
6 to less than 12, n (%)	7 (22.6%)	1 (5.6%)	19 (19.4%)	6 (23.1%)	
12 to less than 24, n (%)	9 (29.0%)	8 (44.4%)	21 (21.4%)	7 (26.9%)	
24 to less than 36, n (%)	2 (6.5%)	0 (0.0%)	8 (8.2%)	1 (3.8%)	
36 to 60, n (%)	7 (22.6%)	3 (16.7%)	10 (10.2%)	1 (3.8%)	
**Diagnosis at Admission**					0.508
Bronchiolitis, n (%)	5 (16.1%)	4 (22.2%)	26 (26.5%)	7 (26.9%)	
Laryngotracheobronchitis, n (%)	1 (3.2%)	0 (0.0%)	1 (1.0%)	0 (0.0%)	
Community-acquired pneumonia, n (%)	25 (80.6%)	14 (77.8%)	65 (66.3%)	19 (73.1%)	
Pertussis-like syndrome, n (%)	0 (0.0%)	0 (0.0%)	6 (6.1%)	0 (0.0%)	
**Underlying Conditions**					
Congenital heart disease, n (%)	0 (0.0%)	3 (16.7%)	1 (1.0%)	4 (15.4%)	0.001
Down syndrome, n (%)	3 (9.7%)	1 (5.6%)	1 (1.0%)	1 (3.8%)	0.135
Bronchopulmonary dysplasia, n (%)	1 (3.2%)	2 (11.1%)	5 (5.1%)	4 (15.4%)	0.209
Immunodeficiency, n (%)	1 (3.2%)	0 (0.0%)	0 (0.0%)	0 (0.0%)	0.203
Asthma, n (%)	2 (6.5%)	4 (22.2%)	5 (5.1%)	0 (0.0%)	0.022
Prematurity (<37 weeks), n (%)	2 (6.5%)	7 (38.9%)	23 (23.5%)	9 (34.6%)	0.028

**Environmental Conditions**					
Breastfeeding, n (%)	25 (80.6%)	16 (88.9%)	83 (84.7%)	20 (76.9%)	0.693
Parental smoking, n (%)	9 (29.0%)	7 (38.9%)	30 (30.6%)	6 (23.1%)	0.729
Gas stove usage, n (%)	31 (100.0%)	17 (94.4%)	93 (94.9%)	25 (96.2%)	0.638
Wood stove usage, n (%)	3 (9.7%)	3 (16.7%)	9 (9.2%)	2 (7.7%)	0.77
Siblings <5 years old, n (%)	8 (25.8%)	7 (38.9%)	40 (40.8%)	5 (19.2%)	0.134
Number of siblings <5 years old (median, IQR)	1.0 (1.0–2.0)	—	1.0 (1.0–1.0)	1.0 (1.0–2.0)	0.03*
Number of household members (median, IQR)	5.0 (4.0–6.25)	5.0 (4.0–6.0)	5.0 (4.0–6.0)	5.0 (4.0–5.75)	0.738*
Daycare attendance, n (%)	2 (6.5%)	0 (0.0%)	3 (3.1%)	4 (15.4%)	0.059
Exposure to similar cases, n (%)	20 (64.5%)	8 (44.4%)	75 (76.5%)	16 (61.5%)	0.035
**Previous Hospitalizations**					
Previous hospitalizations, n (%)	10 (32.3%)	5 (27.8%)	13 (13.3%)	9 (34.6%)	0.027
Number of previous hospitalizations (median, IQR)	1.0 (1.0–1.0)	—	1.0 (1.0–2.0)	1.0 (1.0–2.0)	0.222*
**Severity Indicators**					
Length of hospital stay, days (median, IQR)	6.0 (3.0–8.0)	3.5 (2.0–7.3)	6.0 (3.0–8.0)	4.5 (1.8–7.8)	0.327*
ICU admission, n (%)	2 (6.5%)	0 (0.0%)	2 (2.0%)	1 (3.8%)	0.518
Mechanical ventilation, n (%)	2 (6.5%)	0 (0.0%)	3 (3.1%)	2 (7.7%)	0.502
Death, n (%)	0 (0.0%)	0 (0.0%)	0 (0.0%)	1 (3.8%)	0.128

*Kruskal-Wallis tests. Values are expressed as the medians and interquartile ranges. Abbreviations: IQR, Interquartile Range; RSV, respiratory syncytial virus; hMPV, human metapneumovirus; SARS-CoV-2, severe acute respiratory syndrome coronavirus 2.

Supplementary Table S11. Prevalence of symptoms among pediatric patients with positive PCR results by virus group.

Signs and Symptoms	hMPV (n = 31)	Influenza (n = 18)	RSV (n = 98)	SARS-CoV (n = 26)	p-Value
Fever, n (%)	27 (87.1%)	14 (77.8%)	64 (65.3%)	15 (57.7%)	0.055
Wheezing, n (%)	12 (38.7%)	8 (44.4%)	46 (46.9%)	14 (53.8%)	0.716
Crackles, n (%)	24 (77.4%)	15 (83.3%)	75 (76.5%)	20 (76.9%)	0.939
Respiratory distress, n (%)	31 (100.0%)	17 (94.4%)	97 (99.0%)	26 (100.0%)	0.292
Cyanosis, n (%)	5 (16.1%)	5 (27.8%)	19 (19.4%)	6 (23.1%)	0.769
Cough, n (%)	31 (100.0%)	18 (100.0%)	96 (98.0%)	25 (96.2%)	0.66
Apnea, n (%)	1 (3.2%)	1 (5.6%)	6 (6.1%)	2 (7.7%)	0.904
Rhinorrhea, n (%)	27 (87.1%)	13 (72.2%)	90 (91.8%)	20 (76.9%)	0.053
Nasal congestion, n (%)	27 (87.1%)	17 (94.4%)	83 (84.7%)	21 (80.8%)	0.626
Sneezing, n (%)	16 (51.6%)	12 (66.7%)	60 (61.2%)	14 (53.8%)	0.654
Vomiting, n (%)	15 (48.4%)	7 (38.9%)	33 (33.7%)	9 (34.6%)	0.516
Diarrhea, n (%)	6 (19.4%)	4 (22.2%)	13 (13.3%)	4 (15.4%)	0.72

Abbreviations: RSV, respiratory syncytial virus; hMPV, human metapneumovirus; SARS-CoV-2, severe acute respiratory syndrome coronavirus 2.

Supplementary Table S12. Comparison of RSV positivity and negativity proportions between post-pandemic (2023-2024) and pre-pandemic (2012-2015) seasons.

	Post-pandemic 2023-2024 (n=390)	Pre-pandemic 2012-2015 (n=1,252)	p-Value
RSV Positive, n (%)	160 (41.0% [95% CI: 36.1-46.1])	547 (43.7% [95% CI: 40.9-46.5])	0.353

Supplementary Table S13. Age category comparison between post-pandemic (2023-2024) and pre-pandemic (2012-2015) cohorts of lower respiratory tract infections.

Age Category (months)	Post-pandemic 2023-2024 (n=390)	Pre-pandemic 2012-2015 (n=1,252)	p-Value
Less than 6, n (%)	130 (33.3%)	548 (43.8%)	< 0.001
6 to less than 12, n (%)	79 (20.3%)	336 (26.8%)	0.009
12 to less than 24, n (%)	98 (25.1%)	264 (21.1%)	0.093
24 to less than 36, n (%)	30 (7.7%)	98 (7.8%)	0.931
36 to 60, n (%)	53 (13.6%)	6 (0.5%)	< 0.001

Supplementary Table S14. Age category comparison between post-pandemic (2023-2024) and pre-pandemic (2012-2015) cohorts of lower respiratory tract infections related to respiratory syncytial virus.

Age Category (months)	Post-pandemic RSV 2023-2024 (n=160)	Pre-pandemic RSV 2012-2015 (n=547)	p-Value
Less than 6, n (%)	64 (40.0%)	267 (48.8%)	0.049
6 to less than 12, n (%)	36 (22.5%)	144 (26.3%)	0.328
12 to less than 24, n (%)	30 (18.8%)	98 (17.9%)	0.809
24 to less than 36, n (%)	11 (6.9%)	37 (6.8%)	0.961
36 to 60, n (%)	19 (11.9%)	1 (0.2%)	< 0.001

Abbreviations: RSV, respiratory syncytial virus.
